# Physical and structural characterization of bis-acryl composite resin

**DOI:** 10.1038/s41598-024-58649-9

**Published:** 2024-04-06

**Authors:** Wendy E. Rodríguez-Guardado, Eric M. Rivera-Muñoz, Janeth Serrano-Bello, Marco A. Alvarez-Perez, Rubén A. Domínguez-Pérez, Elias Nahum Salmerón-Valdés, Febe C. Vázquez Vázquez, Osmar A. Chanes-Cuevas, Beatriz Millán-Malo, Carmen L. Peza-Ledesma, Rodrigo Correa-Prado

**Affiliations:** 1 Multidisciplinary Dental Research Laboratory, School of Medicine, Autonomous University of Querétaro, Santiago de Querétaro, Mexico; 2 Centro de Física Aplicada y Tecnología Avanzada, Universidad Nacional Autónoma de México, A.P. 1-1010, Querétaro, 76000 México; 3 Tissue Bioengineering Laboratory, School of Dentistry, National Autonomous University of Mexico, Circuito Exterior s/n, 04510 Mexico City, Mexico; 4 Center for Research and Advanced Studies in Dentistry, Faculty of Dentistry, School of Dentistry, Autonomous University of Mexico State, 50130 Toluca, Mexico; 5 Dental Biomaterials Laboratory, Postgraduate Division, Dental School, National Autonomous University of Mexico, 04510 Mexico City, Mexico

**Keywords:** Dental materials, Provisional restorations, Bis-acryl resins, Fracture resistance, Physical–chemical properties, Chemistry, Engineering, Materials science

## Abstract

During the preparation of fixed prosthesis (including individual bridges and crowns) it is important to select the materials that have the best features and properties to predict a successful clinical treatment. The objective of this study was to determine if the chemical and structural characteristics could cause to increase the fracture resistance, we used four bis-acryl resins Luxatemp, Protemp, Structur and Telio. Three-points bending by Flexural test were performed in ten bars and they were carried out to compare with Anova test. In addition, the bis-acryl resins were analyzed by scanning electron microscopy, to analyze microstructure and morphology and the molecular structure were performed by Infrared Spectroscopy through Attenuated Total Reflectance. A higher flexural strength was found in Luxatemp and Structur with, no significant differences between this study groups. Regarding Protemp and Telio, these study groups showed a lower flexural strength when were compared with Luxatemp and Structur. These results corroborate SEM and ATR analysis because Luxatemp sample showed a regular size particle on the surface and chemically presents a long cross-linkage polymer chain. The presence of CO_3_, SiO_2_ and N–H groups as a fillers particle interacting with OH groups cause a higher flexural strength compared with another groups.

## Introduction

Recently the provisional materials of restoration have been under an exhaustive study in practice for dentistry. The development of innovative materials designed to enhance stabilization, and function for a limited period, must be in accordance with the Glossary of Prosthodontic Terms. Furthermore, any treatment aimed at providing and protecting prepared abutment teeth should always be in search of improving the restoration of occlusal parameters, as well as trying to maintain aesthetic and periodontal health conditions^1–3^.

It is important, therefore, to keep in mind the chemical compounds and properties of provisional restoration as the addition of fine particle size can also enhance polish ability, hardness, smoothness on its surface profile, and color stability to determine its use in different clinical situations^4,5^.

However, there are several risk factors associated with its failure, such as the chewing force that require specific mechanical properties that survive the repeated functional force of the oral environment and pathological disorders induced from parafunctional habits that can compromise the patient´s contentment and comfort^6,7^.

Bis-acryl composites resins were introduced in the 90's and gradually replaced the auto-polymerizing poly (methyl) methacrylate (PMMA) to overcome its negative properties. The Bis-acryl composite resin contains divinyl methacrylate monomers; nevertheless, it has different filler particle loading and chemical composition^8,9^.

Our main aim was to characterize different Bis-acryl composite resin using mechanical, microscopic, and spectroscopic analysis in order to identify the chemical and structural characteristics of a provisional material, which could cause a better fracture resistance by flexural test on three-points, using four types of bis-acryls (Luxatemp, Protemp, Structur Premium, and Telio).

## Results

### Mechanical properties

The characterization of the mechanical properties of the four bis-acrylic materials were performed by obtaining the values of the fracture resistance by flexural test (Table 1). The values showed that Luxatemp has the higher flexural strength (211.44 ± 23.31 MPa), followed by Structur (207.33 ± 17.26 MPa). Furthermore, Protemp (173.57 ± 14.10 MPa) and Telio (152.00 ± 25.94 MPa) showed less flexural strength in comparison to the Luxatemp and Structur (Table 1). In Table 2, ANOVA test showed statistically significant differences between study groups regarding to flexural strength of four bis-acryl resins analyzed in this study with a p value of 0.0001. A post Hoc Tukey analysis was developed to identify between which groups these differences occurred. As can be seen in Table 3, the statistically significant differences were observed when compared Luxatemp with Protemp and Telio (p = 0.001 and p = 0.0001) respectively. These differences were also observed when comparing Structur group with Protemp and Telio (p = 0.004 and p = 0.0001) respectively. However, this mechanical behavior is related to the different ultrastructural surface morphology and the filler material as showed differences in the four bis-acrylic materials by SEM.

**Table 1:** Descriptive results of study groups and Shapiro Wilk test.

	Structur	Luxatemp	Telio	Protemp
Mean ± SD	207.33 ± 17.26	211.44 ± 23.31	152.00 ± 25.94	173.56 ± 14.09
SW test p value	0.266	0.222	0.118	0.877

SD: Standard Deviation, p value ≤ 0.05: significant differences, SW: Shapiro Wilk.

**Table 2:** ANOVA test.

	Sum of squares	df	quadratic mean	F value	p value
Between groups	24,131,098	3	8043,699	18,780	0.0001
Within groups	15,418,931	36	428,304		
Total	39,550,029	39			

df: degrees of freedom, p value ≤ 0.05: significant differences.

**Table 3:** Tukey test to compare differences between study groups.

Intergroup comparison	Mean difference	p value
Luxatemp-Structur	4.11400	0.970
Luxatemp-Telio	59.44500	0.0001*
Luxatemp-Protemp	37.88200	0.001*
Structur-Telio	55.33100	0.0001*
Structur-Protemp	33.76800	0.004*
Telio-Protemp	21.56300	0.110

*p value ≤ 0.05.

### SEM analysis

**Figure 1:**
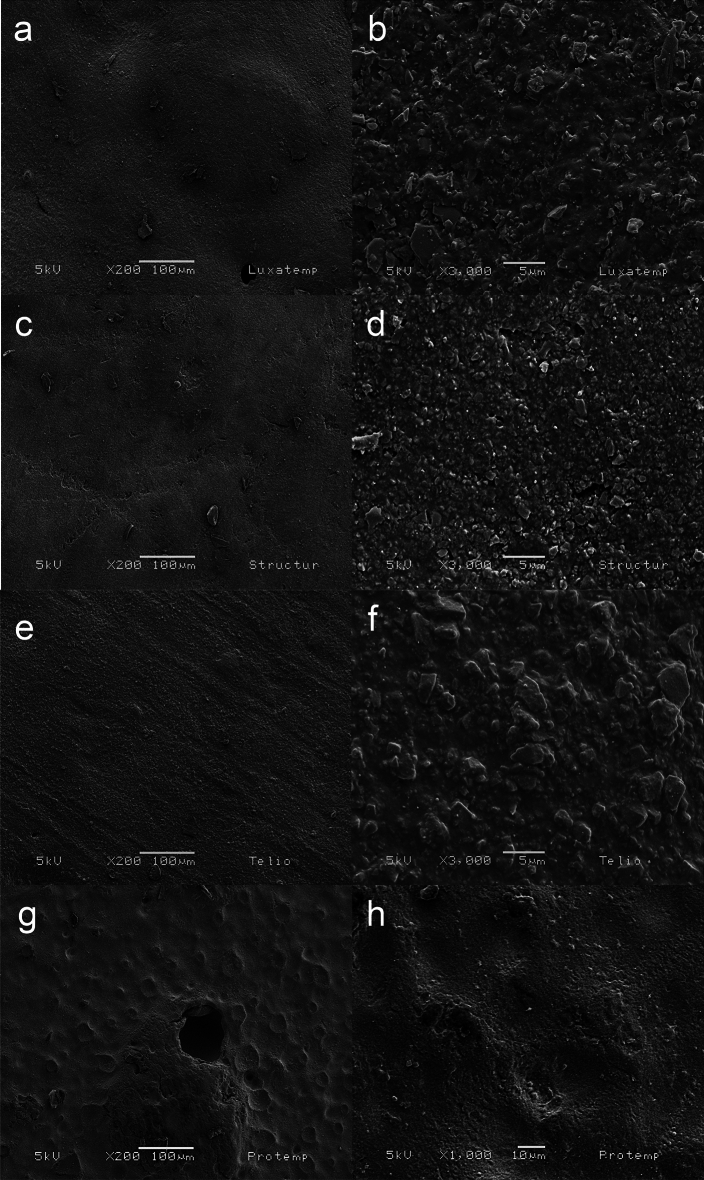
Scanining electron microscope (SEM) images of bis-acryl composite resins of Luxatemp (**a**,**b**) Structur (**c**,**d**) Telio (**e**,**f**) and Protemp (**g**,**h**).

The analysis of the ultrastructural surface morphology of the four bis-acrylic materials showed an irregular surface with different particle size (Fig. 1). For Luxatemp bis-acrylic SEM analysis showed a micro particles approximately of 5 µm to 10 µm distributed on the irregular surface (Fig. 1a and b). The Structur bis-acrylic showed a similar irregular surface topography with different shapes and overlapping particles that has a smaller than 5 µm in size (Fig. 1c and d). The Telio bis-acrylic images revealed some fine wrinkles observed on the surface with greater amounts of filler particles with size of 1–6 µm, which were more densely and attached to the surface (Fig. 1e and f). Finally, the Protemp bis-acrylic showed an irregular surface with craters and round hole-likes with size of 20–40 µm and particles that surround them (Fig. 1g and h).

### FTIR analysis

**Figure 2:**
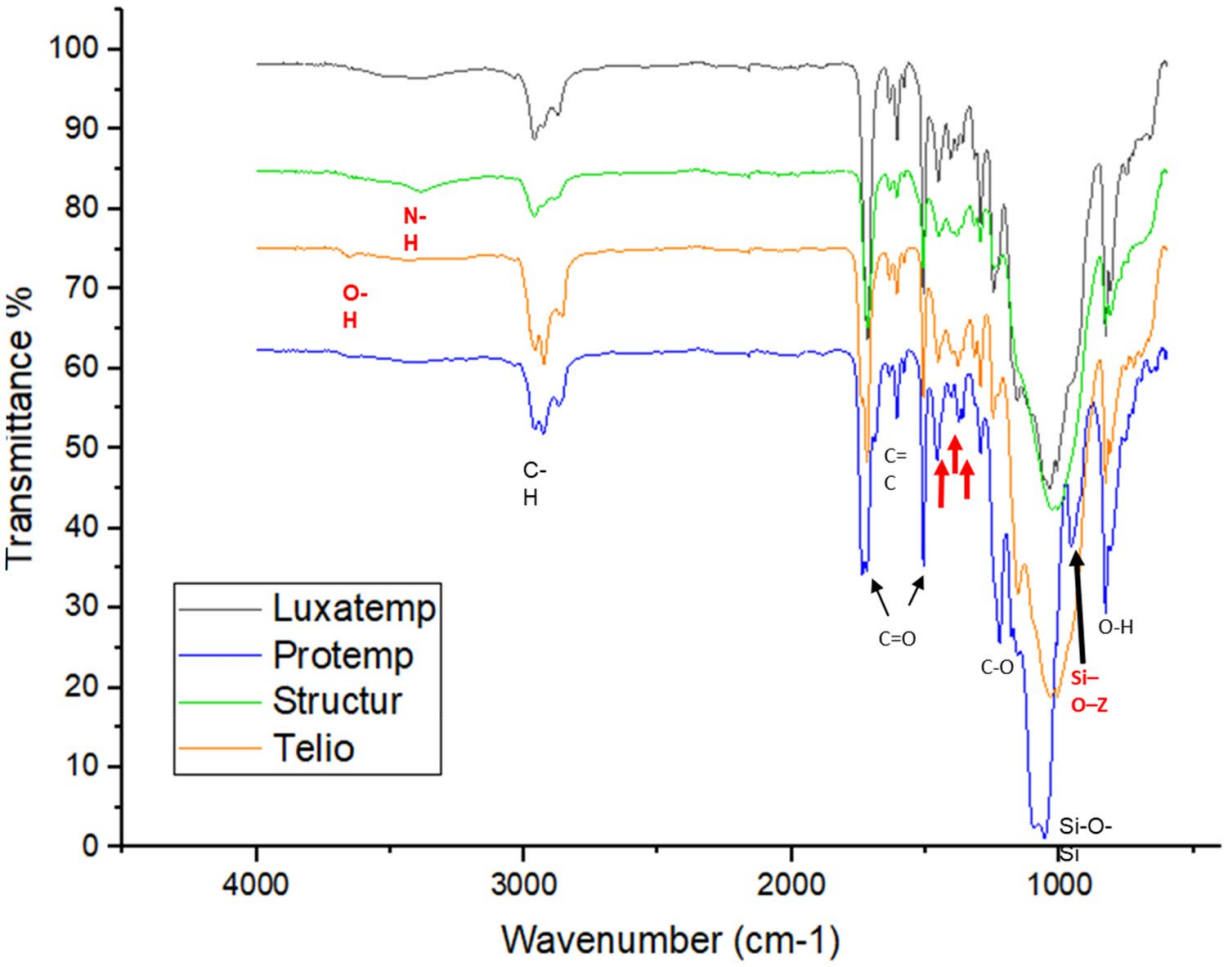
Infrared spectra for bis-acryl resins.

The molecular structure and the functional groups presented in the four bis-acrylic materials were analyzed by FTIR spectroscopy (Fig. 2). The vibration bands at 3600–3400 cm^−1^ are related to the N–H groups in the Structur and Telio materials. In the range of the 2970–2840 cm^−1^ is related to asymmetric and symmetric stretching of methylene group CH_2_ vibrations in the Structur, Telio, Protemp, and Luxatemp materials. Moreover, the vibrations at 1740–1710 cm^−1^, 1640–1600 cm^−1^, and a double band in the region of 1510–1450 cm^−1^ is attributed to C=O of Poly (methyl methacrylate) for the four bis-acrylic materials. The C–O stretching vibration peaks were at 1260–1230 cm^−1^, and 1160 cm^−1^. The asymmetric Si–O–Si stretching vibrations was observed at 1090 cm^−1^, and 1020 cm^−1^ meanwhile the O–H deformation vibration peak was at 820–830 cm^−1^ when the hydroxyl group was bonded to a silicon atom. Finally, there were different identified weak bands between 1460 and 1350 cm^−1^ assigned to pigment materials, stabilizers, and filler particles.

## Discussion

Recently, several manufactures and modifications of new materials have been introduced, but there is not much research on the mechanical performance and its relationship with the ultrastructural characteristics and chemical compounds to provide them resistance available of these bis-acryl composites. Our results were similar to the last research of Luxatemp and other three bis-acrylic (Provipont, Protemp 3 Garant, and Integrity) resins demonstrated significantly superior flexural strength over traditional methacrylate resins. Such results suggest that this behavior is due to dysfunctional and capacity of cross-linking with another monomer chain imparting strength and toughness to the materials^5^. There was a condition to be considered, as the time to exposure to water because in previous reports it has been demonstrated that a highly significant decrease in flexural strength was seen for the bis-acrylic materials (Protemp 4, Cooltemp natural, and Luxatemp Fluorescence) from 24 h to 8 days, but there was no difference at 8 days^10^, it is possibly attributable to the excessive water uptake, which can promote breakdown causing a filler matrix debonding, this can spread polymer chains apart and facilitate slippage between chains^11^.

At the other extreme, temporary crowns were produced by a direct fabrication methods, by using bis-acrylic composites (Structur Premium, Takilon, System c & b II, and Acrytemp) and they were subjected to sterilization wrapping at room temperature to be measured and determined the fracture strength, which showed that Structur Premium obtained the highest values in comparison to the other bis-acrylic materials, and they concluded that different chemical composition could be the cause for difference in fracture strength being difunctional materials, thus, they are capable of cross-linking with another monomer chain^12^.

Nevertheless, our study demonstrated that the images obtained of Protemp sample by SEM, showed hole-likes distributed over the surface and craters-likes which it was not possible to establish a calculating in the depth, hence, we suggest that this decreases the fracture resistance and possibly in a clinical condition act as a bacterial reservoir.

Telio sample showed the larger particle size (> 3 µm), this provides for crack propagation within the sample to occur primarily through the polymer matrix between filler particles^13^. The manufacturer indicates an inorganic filler is the cause of failure to the flexural testing, and it is not able to stop the fracture.

The surface of Luxatemp and Structur were similar in other study moreover the failure analysis was performed by biaxial flexure test showing an increase in value of Structur after 15 and 30 days of storage compared with 7 days^14^. They assumed that water storage yielded a more brittle behavior, this was due to the crack propagation within bis-acryl materials that occurs primarily through the polymer matrix between filler particles^13^, and it may interfere in the mechanical performance principality for the water sorption and swelling that could affects the overall polymer strength^15^.

Inorganic Filler particles related to Silica (Si) were observed in FTIR spectra at about 1090–1020 cm^−1^ and a possible interaction at 820–830 cm^−1^ with the O–H^16,17^, in Protemp sample showed a weak signal at 954 cm^−1^ corresponding to the Si–O–Z cross-linkage^18^, these zirconium particles present in Protemp sample to have shown a decrease in the fracture strength by flexural test on three-points. Our outcome was different from other prior studies^19–22^, where Protemp shows higher values using different.

In the present study it still was necessary to promote more variables that should be assessed and that emulating conditions of the oral environment as all the above-mentioned studies. There were considerable variations in length and width between all samples at 1460–1350 cm^−1^; these bands were accountable of the resistance shown by Structur and Luxatemp samples, these bands usually correspond to cross-linkage between Carbonates^23^, (CO_3_), Barium sulphate (BaSO_4_), Silicon Dioxide (SiO_2_), Aluminum Silicate (Al_2_SiO_5_), Barium Silicate (BaO_3_Si) and Silicon Bromide (Br_4_Si)^14–24^.

The presence a band showed at 3380 cm^−1^ and 3660 cm^−1^ in Structur and Telio samples respectively confirm the typical absorptions of OH group, which has been the cause for the decrease in the fracture resistance as it has been reported in other study^11^.

According to the findings of our study, Luxatemp was the bis-acryl resin with the highest flexural strength although Structur showed a similar flexural strength with significant difference when was compared to Protemp and Telio. These outcomes are related to the filling particles, mainly zirconium and silica, which could cause the average pore size and irregular surface of the polymer chains as indicated by SEM. Furthermore, the absorption of OH can interact with the filler matrix promoting, consequently, the decrease in the polymer strength by interactions with CO_3_, SiO_2_ and N–H groups. Nonetheless, it is necessary to carry out more studies with conditions that emulate the clinical and oral environment.

## Materials and methods

### Preparation of specimens

This study was designed to characterize and compare four commercial bis-acryl composite resins: (1) Luxatemp (DMG), (2) Protemp (3M ESPE), (3) Structur Premium (VOCO), and (4) Telio (Ivoclar Vivadent) using SEM, Infra-red and mechanical analysis. Paste and catalyst pastes of each bis-acryl composite resin were mixed with dispensing guns and automix syringes and placed into molds according to norm 27 ANSI/ADA No. 27. All samples used for the assay previously rinsed with 70% ethanol and stored in double distilled water by 14 days at 37 °C.

### Properties characterization of bis-acrylic specimens

The ultrastructural surface morphology of the four bis-acrylic materials was made by a SEM microscopy (JSM-6060LV), using an acceleration voltage at 5 kV with secondary electrons were performed. All samples for SEM analysis were sputtering coated (EMS 559) with a golden thin film. On the other hand, the physicochemical properties of the four bis-acrylic materials were evaluated with a FTIR spectrophotometer (Pekin Elmer) using a diamond ZnSe crystal plate. Forty scans for each spectrum were collected acquiring 1 scan/s at 5 cm^−1^ resolution in the wavenumber range of 500–4500 cm^−1^. Lastly the fracture resistance by flexural test on three-points mechanical assay were performed ten specimens from each bis-acrylic material were used under the norm 27 ANSI/ADA No. 27. The flexure strength was performed on a computer-controlled universal testing machine (CMS Metrology, Model WDW-5Y, Querétaro, Mexico) by means of the three-point bend test. Each specimen was mounted with its edges equidistant from the midline of the holder. The load was applied at a crosshead speed of 0.75 mm/min until its fracture. The data were collected in Newtons and converted to megapascals (MPa) according to the following equation: Flexure strength = 3FL/(2BH^2^), where the maximum load was represented by F; L was the distance between supports (mm); B was the width of the specimen (mm); and H was the height (mm).

### Statistical analyses

The flexural strength results are expressed as means and standard deviation and were analyzed with the statistical software SPSS Version 26 through descriptive and inferential statistics using Shapiro Wilk, and ANOVA test with post-hoc Tukey. Statistical significance was set at *p* ˂ 0.05.

## Data Availability

The data presented in the study are available for publication.
